# Study on Dynamic Characteristics of Resilient Mount Under Preload

**DOI:** 10.3390/ma17205096

**Published:** 2024-10-18

**Authors:** Sung-Ju Park, Byoungjae Park, Joo-Yeob Lee, Yun-Ho Shin, Chae-Lim Jeong, Sung-Jae Kim, Kookhyun Kim

**Affiliations:** 1 School of Electrical & Control Engineering, Tongmyong University, Busan 48520, Republic of Korea; parksj0314@tu.ac.kr (S.-J.P.); cljeong@tu.ac.kr (C.-L.J.); 2 Open Grid Laboratory, Tongmyong University, Busan 48520, Republic of Korea; shinyh77@pusan.ac.kr; 3 Korea Research Institute of Ships and Ocean Engineering, Daejeon 34103, Republic of Korea; 4 Global Core Research Center for Ships and Offshore Plants (GCRC-SOP), Pusan National University, Busan 46241, Republic of Korea; hermedo@pusan.ac.kr; 5 Department of Naval Architecture and Ocean Engineering, Pusan National University, Busan 46241, Republic of Korea; 6 Fisheries Engineering Division, National Institute of Fisheries Science, Busan 46083, Republic of Korea; sjkim8@korea.kr

**Keywords:** resilient mount, hyperelastic material, driving point dynamic stiffness, Yeoh model, static and dynamic analysis

## Abstract

Resilient mounts are essential for anti-vibration and shock absorption applications, making accurate predictions of their static and dynamic behaviors critical for effective design and mechanical performance. This study investigates static and dynamic characteristics of resilient mounts to predict their effects. Tension, compression, and shear tests were performed under quasi-static loading conditions to obtain stress-strain cycle curves. This study includes a review of the Yeoh hyperelastic model, which consists of three parameters, and discusses the calibration of these parameters to describe the hyperelastic material behavior. The parameters were validated through numerical analysis by comparing them with experimental results from quasi-static tests on the resilient mount. The dynamic behavior was further analyzed using modal analysis and frequency response simulations under various preload conditions. Results show that increasing preload significantly shifts the transmissibility curves and resonance peaks to lower frequencies. This study offers valuable insights into static and dynamic characteristics of resilient mounts, contributing to the design and optimization of vibration isolation systems for naval applications.

## 1. Introduction

Resilient mounts play a crucial role in vibration damping within mechanical systems, including naval ship applications. These mounts are essential for predicting and controlling the transmission of vibrational energy from engines to ship structures. Material properties and working environment factors such as temperature, preloading, and frequency significantly influence the mechanical behavior of resilient mounts. Accurate evaluation of the system through simulations based on precise material modeling is necessary to describe this behavior accurately.

To identify static and dynamic characteristics of rubber mounts, researchers have studied the materials, mount structure, and preload to facilitate their development and application. Kari [1] proposed an improved measurement method for determining the dynamic transfer stiffness of vibration isolators in multiple degrees of freedom up to 1000 Hz, including techniques to increase accuracy, such as using an improved excitation arrangement, source correlation, stepped sine excitation, and effective mass calibration. Amin et al. [2] proposed an improved hyperelasticity model and parameter identification scheme for representing the rate-independent monotonic behavior of natural rubber and high-damping rubber in compression and shear regions, implemented in finite element analysis and verified through experiments and numerical simulations. Sasso et al. [3] conducted equi-biaxial, uniaxial, and planar tension tests to calibrate and validate hyperelastic models, comparing the results with static simulations for mechanical tests. Lee et al. [4] presented a method for predicting the dynamic stiffness of rubber components under various preload conditions by using the static and dynamic stiffness of rubber material specimens and the static stiffness of the rubber component. Ritto et al. [5] presented a Bayesian approach to identifying parameters and ranking different hyperelastic material models for simple and pure shear, finding that the Mooney–Rivlin model performed best for pure shear while the 2-term Ogden model was best for simple shear.

Akyüz et al. [6] investigated the effects of different NR (Natural Rubber)/SBR (Styrene Butadiene Rubber) ratios on the mechanical properties and performance of anti-vibration bushings, finding that increasing the NR content up to 70% improved dispersion, tensile strength, and durability while reducing hysteresis. Kamaruddin et al. [7] investigated the mechanical properties of hybrid-filled natural rubber, including static stiffness, hysteresis loss ratios, stress relaxation responses, dynamic stiffness, phase angles, and damping ratios through sample testing. Luo et al. [8] presented an energy dissipation approach for predicting the complete loading-unloading response of rubber anti-vibration components under quasi-static conditions and a natural frequency region (NFR) method for simulating dynamic impact response, with experimental validation on an industrial rubber mount. Íñiguez-Macedo et al. [9] conducted standardized tests and investigated the error of various hyperelastic models through experiments. Hazra et al. [10] discussed the rubber shape of motor mounts to avoid high-frequency resonance. Wu et al. [11] presented the high-frequency dynamic characteristics of rubber mounts under different preloads. Moon et al. [12,13] developed an inertia-type hybrid mount combining a rubber mount and piezo stack actuator for vibration isolation of shipboard equipment, which demonstrated improved performance over passive mounts in experimental evaluations. Moon et al. [14] conducted performance tests on mounts for shipboard equipment in naval ships, adhering to military standards. Gong et al. [15] presented a nonlinear model for rubber elements in high-speed railway vehicle suspensions that accounts for temperature, frequency, and amplitude effects and used it to analyze how temperature changes impact the coupled vibrations of the train car body and under-chassis equipment, finding that low temperatures significantly increase rubber stiffness and degrade ride quality. Fragasso et al. [16] optimized the coefficients of the Yeoh hyperelastic material model for a rubber-resilient mount used in marine diesel engines by comparing experimental compression test data to finite element simulations using response surface methodology. Fragasso et al. [17] presented an experimental and numerical methodology for characterizing the dynamic properties of resilient mounts used to isolate marine diesel engines. This involves measuring transmissibility curves, finite element modeling, and optimizing frequency-dependent damping ratios to match experimental data. Bonisoli et al. [18] presented an enhanced design procedure for automotive wheels, taking into account elastoplastic material properties and manufacturing process parameters to predict the local stiffening effect between the rim and disk due to residual stress variations. Emminger et al. [19] discussed the determination of hyperelastic material parameters and presented a numerical study of dampers made from thermoplastic polyurethane (TPU) and polydimethylsiloxane (PDMS), focusing on their mechanical behavior and applications in damping.

This study focuses on characterizing and modeling hyperelastic behavior in rubber materials used for resilient mounts in naval ship applications. Three types of standardized tests were conducted to investigate the static mechanical properties of the rubber. Yeoh’s constitutive material model was fitted using the test data and subsequently verified through finite element analysis (FEA) of resilient mount tests. Additionally, dynamic analysis was performed to investigate the characteristics of resilient mounts under forced preloading conditions. This work aims to develop a general procedure for investigating static and dynamic characteristics of resilient mounts for practical applications using an FEA approach.

## 2. Parameters Identification of Hyperelastic Material

The rubber material used in a wide variety of structural applications for anti-vibration and soundproofing can exhibit non-linear elastic behavior as a hyperelastic material under large deformations. Accurate material constitutive model information is essential for improving the accuracy of finite element static analysis for hyperelastic material. Quasi-static tests at room temperature were conducted to measure the mechanical properties needed for calibrating hyperelastic constitutive model parameters. This section briefly introduces the hyperelastic model and the calibration process for material parameters.

### 2.1. Yeoh Model

The elastic deformation for rubber material can be described in terms of the elastic strain energy density W, which represents an invariant of the right Cauchy–Green strain tensor. This concept is widely used to describe the elastic deformation of a homogeneous isotropic elastic body. It is given by:

(1) $$W=\frac{1}{2}Nk\theta \left(\lambda_{1}^{2}+\lambda_{2}^{2}+\lambda_{3}^{2}-3\right)$$

where N is the number of network chains per unit volume, k is Boltzmann’s constant, and θ is the absolute temperature. $\lambda_{i}\left(i=1,2,3\right)$ are the principal stretch ratios, defined as the extension ratio.

For an incompressible material, Mooney–Rivlin [20] used this to define an approximate expression in terms of the polynomial form:

(2) $$W=\sum_{i,j=0}^{\infty }C_{ij}{\left(I_{1}-3\right)}^{i}{\left(I_{2}-3\right)}^{j}$$

$C_{ij}$ is material constants. $I_{1}$ and $I_{2}$ are the first and second invariants of the stretch tensor.

The Yeoh model [21,22] can be expressed by setting i=1,2,3 and j=0 in the polynomial form. This simplifies the expression to:

(3) $$W=\sum_{i=1}^{3}C_{i0}{\left(I_{1}-3\right)}^{i}$$

(4) $$W=C_{10}\left(I_{1}-3\right)+C_{20}{\left(I_{1}-3\right)}^{2}+C_{30}{\left(I_{1}-3\right)}^{3}$$

$C_{10}$, $C_{20}$, and $C_{30}$ were defined as half of the initial shear modulus, the softening parameter, and the hardening parameter, respectively. 

The Yeoh model is suitable for a wide range of deformations and can predict stress-strain behavior across various modes with less data, offering simplicity, fewer required parameters, and improved predictive ability, making it convenient for material constant calibration [23,24]. In this study, the Yeoh constitutive model is utilized in finite element analysis to simulate the static force-deformation characteristics of hyperelastic materials, such as rubber, which exhibit nonlinear elastic responses under large strains.

### 2.2. Quasi-Static Tests

**Figure 1:**
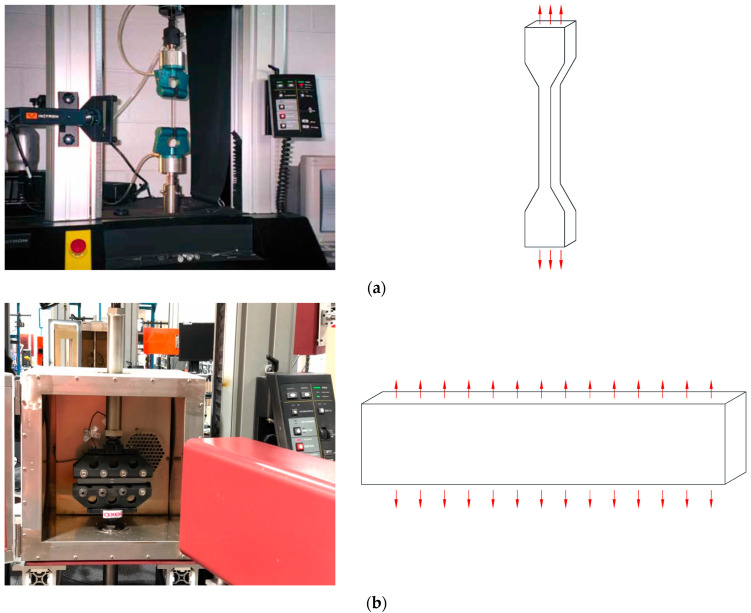

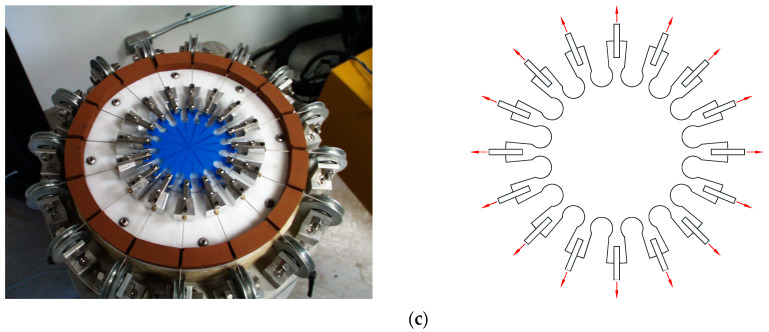
The set-up of experiments (source: https://www.axelproducts.com/ (accessed on 15 October 2024)) and schematic illustrations of deformation modes (**a**) uniaxial tension, (**b**) planar tension, and (**c**) equi-biaxial tension.

**Figure 2:**
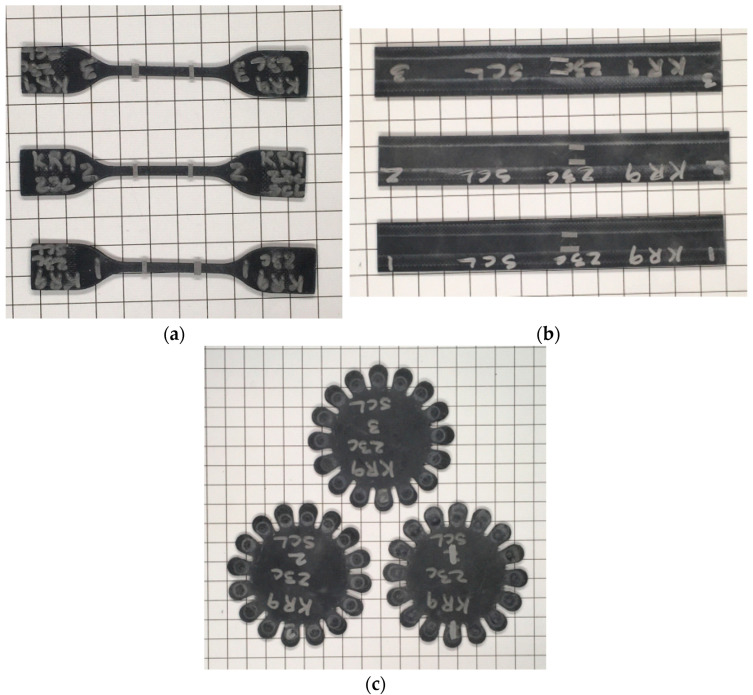
The photo of specimens (**a**) uniaxial tension, (**b**) planar tension, and (**c**) equi-biaxial tension.

**Figure 3:**
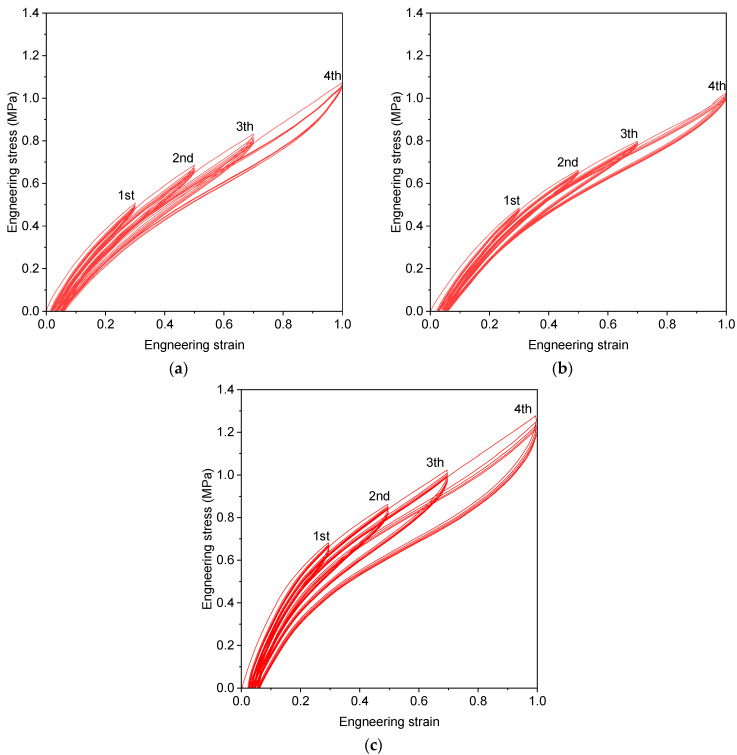
Stress-strain curve under four strain levels (**a**) uniaxial tension, (**b**) planar tension, and (**c**) equi-biaxial tension.

The uniaxial tension, biaxial tension, and compression experiments were conducted to calibrate the material constitutive model of the rubber element in the resilient mount. The hysteresis loading test is conducted under four strain levels (30%, 50%, 70%, and 100%) for uniaxial tension (UT), planar tension (PT), and equi-biaxial tension (EBT) tests. The experiments were conducted by Axel Products, Inc. (Ann Arbor, MI, USA). Figure 1 depicts the set-up of experiments and schematic illustrations of the deformation mode. Figure 2 shows the photos of the specimens. The test procedure followed the ASTM D412-16 standard [25]. The tension specimen was type D, with a thickness of 2.0 mm. The flat tensile test is conducted using a uniaxial load with no restrictions in the transverse direction, which is why it is also referred to as a pure shear test. The planar tension specimen has an aspect ratio of approximately 10:1. Generally, the compression test is less accurate due to friction and interference between the specimen and the testing equipment. Therefore, data are obtained through a biaxial tension test, which is considered equivalent to the compression test. The equi-biaxial tension specimen was specifically designed to maintain an equal biaxial strain state by radially stretching a circular disc. When an elastomer is subjected to cyclic deformation, it undergoes stress softening, a phenomenon known as the Mullins Effect. The most significant softening occurs during the initial cycle, with subsequent cycles showing a diminishing softening rate. Hysteresis loading was applied five times at each strain level to mitigate the Mullins Effect sufficiently in the rubber material. The test speed was eight mm/min at room temperature. Figure 3 presents the engineering strain-engineering stress cycle loading curves for each stretch level (30%, 50%, 70%, and 100%).

### 2.3. Calibration of Yeoh Model Parameter

**Figure 4:**
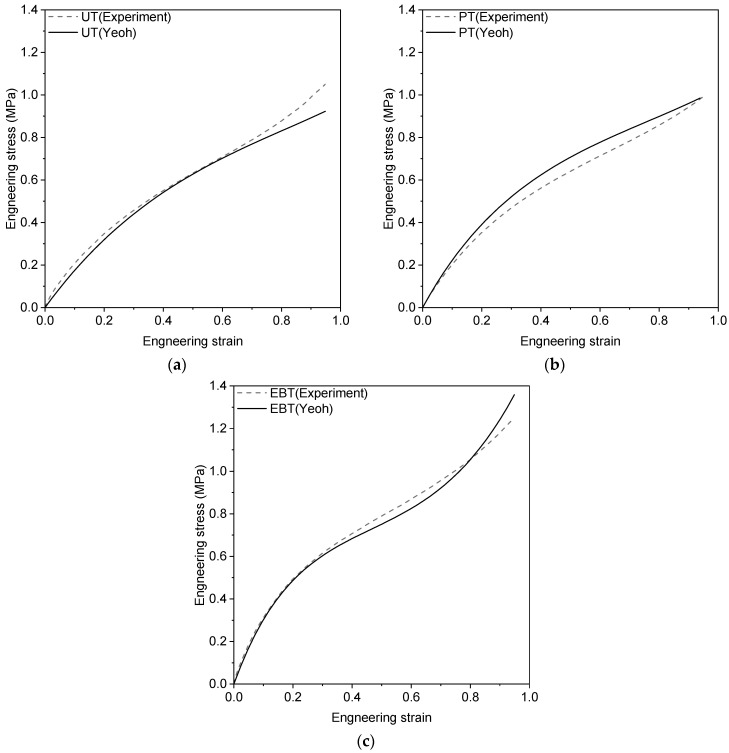
Experimental and fitted results of Yeoh model (**a**) uniaxial tension, (**b**) planar tension, and (**c**) equi-biaxial tension.

Yeoh model parameters were identified by fitting the last loading curve to each set of experimental data using the least square method. Figure 4 shows the fitting results, and the constitutive parameters were $C_{10}=320,520.65\;\mathrm{Pa}$, $C_{20}=-23,411.40\;\mathrm{Pa}$, and $C_{30}=3874.44\;\mathrm{Pa}$. Yeoh model relies exclusively on the first invariant, which facilitates the optimization of the model parameters. The model has noted that the shear modulus significantly decreases at low strains, a behavior that could not be accurately predicted by existing hyperelastic models at that time [21,22]. In the case of the resilient mount, it was concluded that the Yeoh model was sufficient for this analysis, as vertical loading (both tensile and compressive) was the predominant factor influencing the results. The optimal values for the material constitutive coefficients of the rubber element were further validated by comparing them with the results from resilient mount tests.

## 3. Validation of Static Mechanical Behavior

### 3.1. Description of Static Test for Resilient Mount

**Figure 5:**
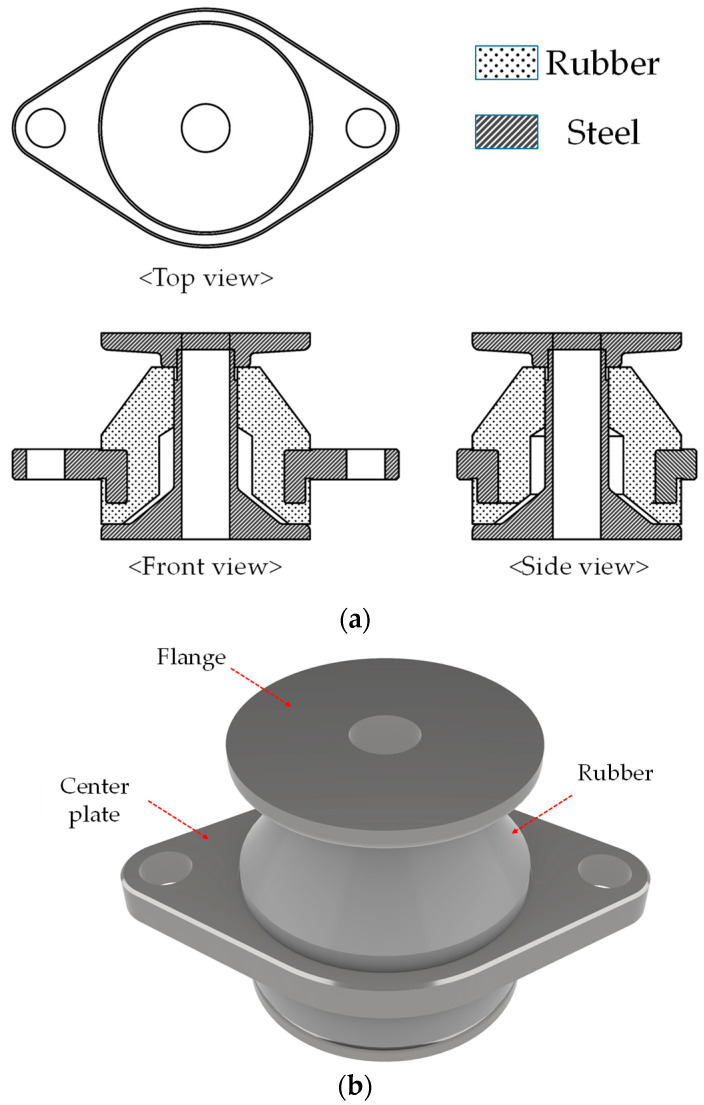
The resilient mount SC-7K450 (**a**) 2D drawing (**b**) 3D model.

**Figure 6:**
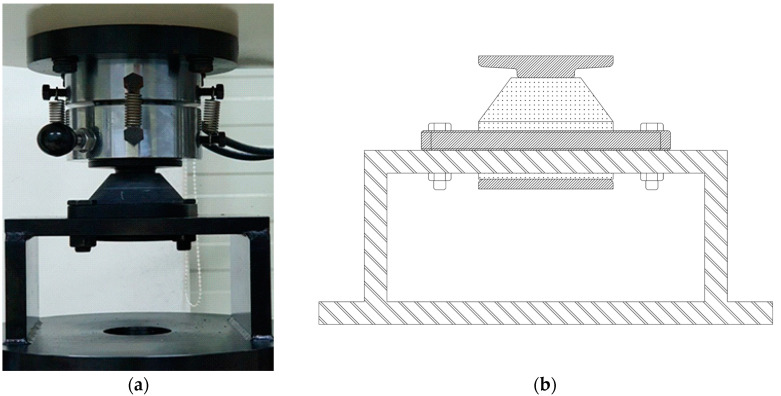
Setup of quasi-static test of resilient mount (**a**) photo (**b**) schematic diagram.

The 2D drawing and 3D model of the resilient mount, which meets the MIL-M-17508F standard [26] and is used for validation, are illustrated in Figure 5. The resilient mount consists of two steel parts (a flange and a center plate) and a rubber element. The experiment was designed to induce large deformations through both compression and tension states. The experimental setup, including the specimen and jig, is presented in Figure 6. The test speed was eight mm/min at room temperature. The compression and tension tests were conducted in two steps. In the first step, three loading and unloading cycles were applied to induce stress-softening up to 39,128.53 N. In the second step, the force and displacement curves were measured with the load applied up to 53,348.17 N.

### 3.2. Finite Element Analysis

**Figure 7:**
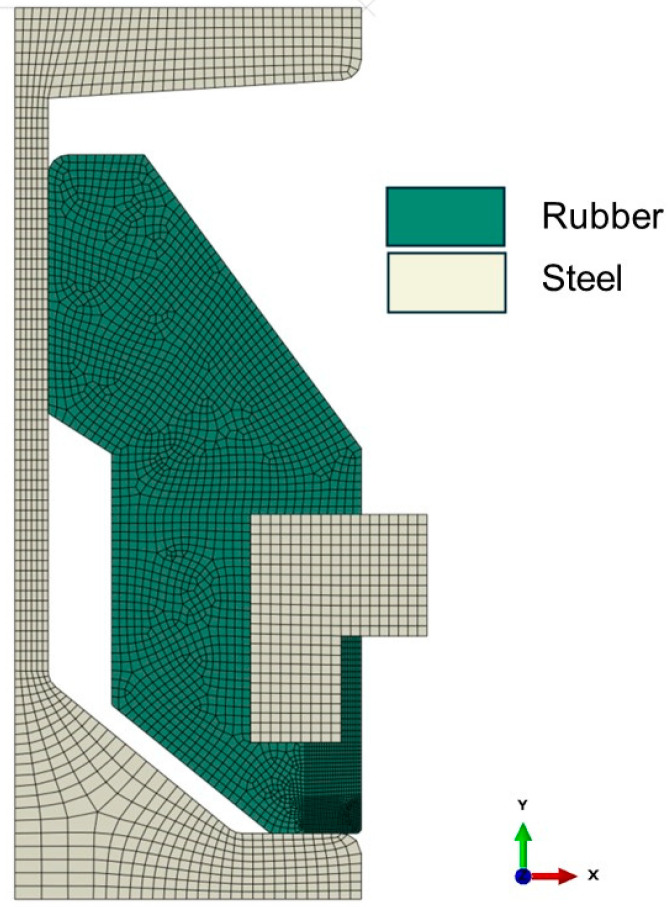
Finite element mode of the resilient mount.

The simulation for the resilient was performed using the commercial finite element program Abaqus. The geometry of the finite element (FE) model is illustrated in Figure 7. The FE model was created using axisymmetric reduced integration hybrid elements (CAX3RH and CAX4RH), which are hybrid elements designed for compressible properties in Abaqus, to discretize the rubber part. In contrast, the steel part was modeled using 4-node axisymmetric reduced integration elements (CAX3R and CAX4R). The mesh size was determined through mesh convergence tests. A contact condition was implemented between the steel and rubber parts using the surface-to-surface contact option with frictionless interaction. The steel part was defined as the master surface, while the rubber part was defined as the slave surface. The mechanical contact properties were set using a hard contact model with zero clearance, ensuring no penetration in the normal direction. This setup was designed to minimize surface penetration at constraint locations, providing an accurate simulation of the contact behavior. The node at the bottom surface was fully constrained. The jig was modeled using axisymmetric rigid elements (RAX2) to apply a forcing displacement on the top surface. The identified Yeoh model was used to define the material property of the rubber part. Young’s modulus, density, and Poisson’s ratio of steel part were 210,000 MPa, 7850 kg/m^3^, and 0.3.

**Figure 8:**
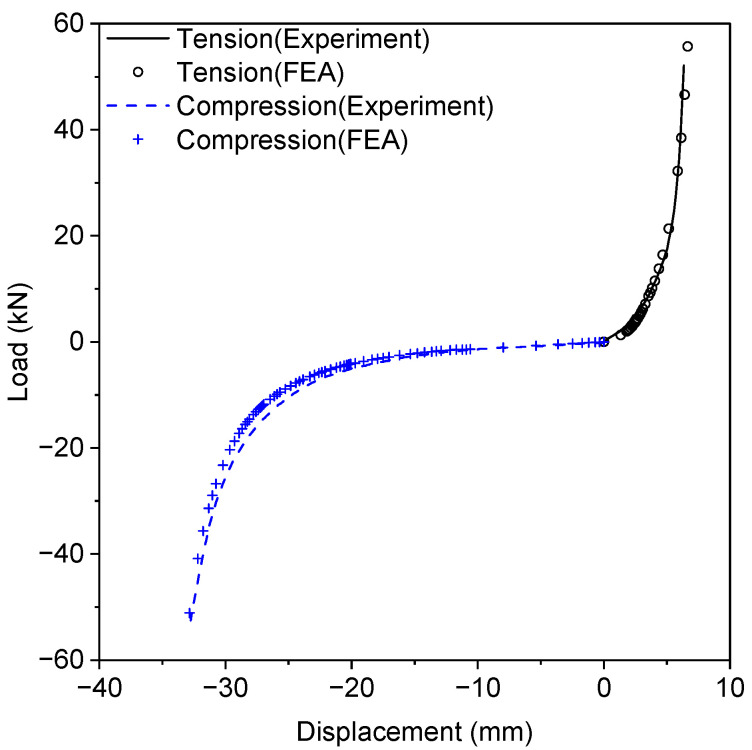
The comparison of stress-strain curve between test and numerical results of resilient mount tests.

Figure 8 illustrates a comparison of the force-displacement curves between experimental and numerical results. Table 1 presents the strain energy density $\left(U_{0}\right)$, which is represented by the area under the stress $\left(\sigma \right)$-strain $\left(\varepsilon \right)$ curve as follows:

(5) $$U_{0}=\int_{0}^{\varepsilon }\sigma d\varepsilon$$

The presented hyperelastic model shows good agreement with the experimental results.

## 4. Dynamic Characterization of Resilient Mount

### 4.1. Modal Behavior of the Resilient Mount Parts

**Figure 9:**
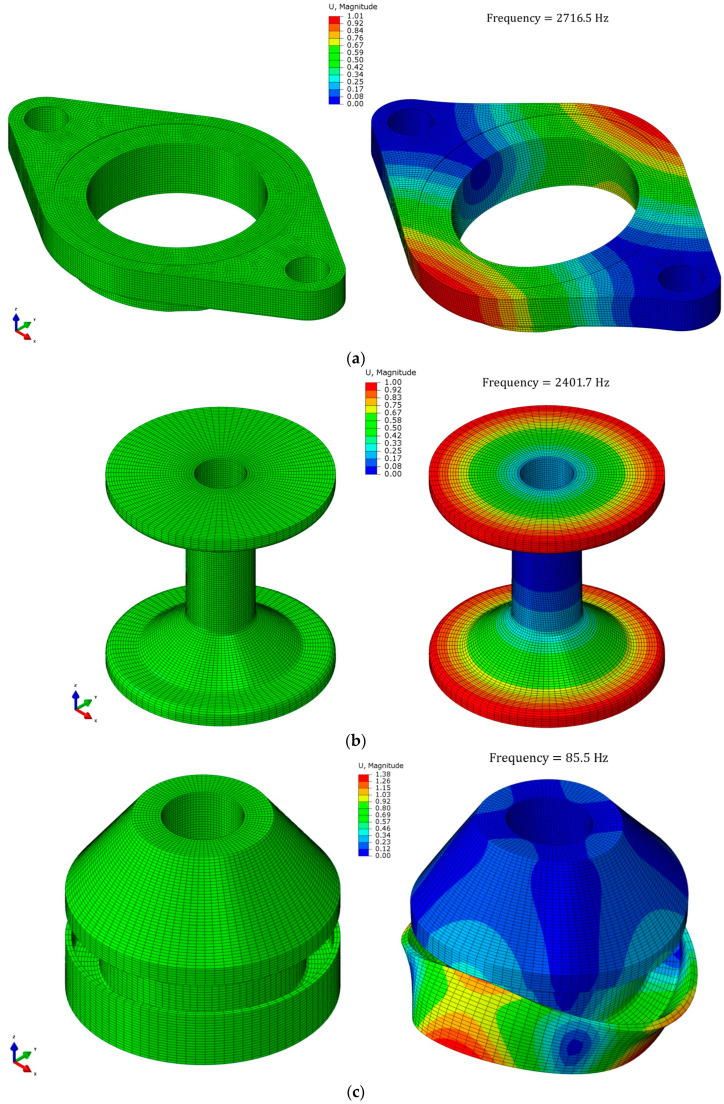
Results of modal analysis (**a**) center plate, (**b**) flange, and (**c**) rubber.

Modal analyses for the three components (center plate, flange, and rubber) were conducted using numerical methods to analyze the dynamic characteristics of the resilient mount. The translational and rotational displacement constraints of the center plate were applied at the two bolted joints, while the flange and rubber were subjected to free boundary conditions. Figure 9 shows the FE model of the three parts (center plate, flange, and rubber element) and mode shapes at their fundamental frequencies. The frequency of the rubber element was less than 100 Hz, while the frequencies of the steel parts were over 2000 Hz.

### 4.2. Dynamic Simulation for the Resilient Mount

**Figure 10:**
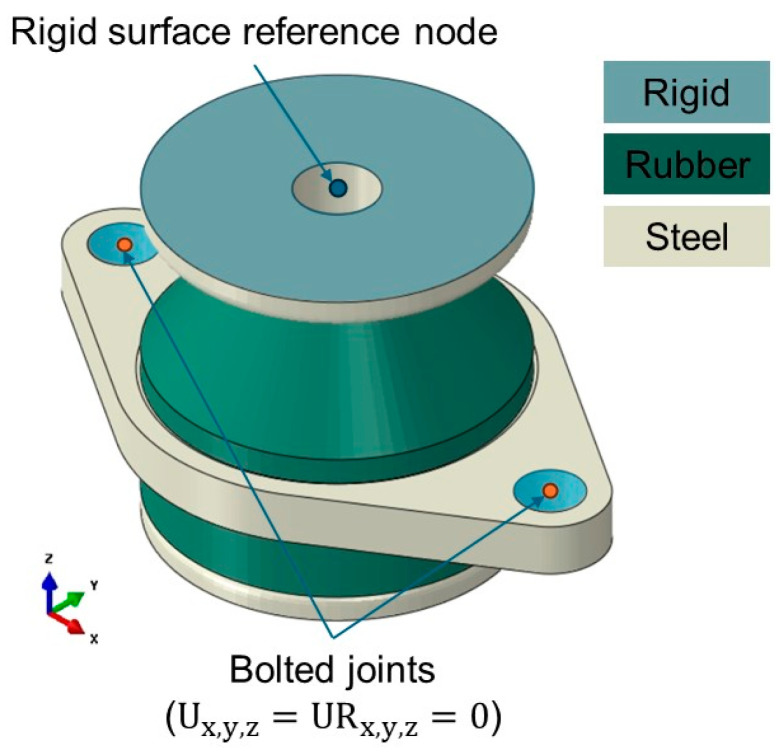
FE model for dynamic analysis.

**Figure 11:**
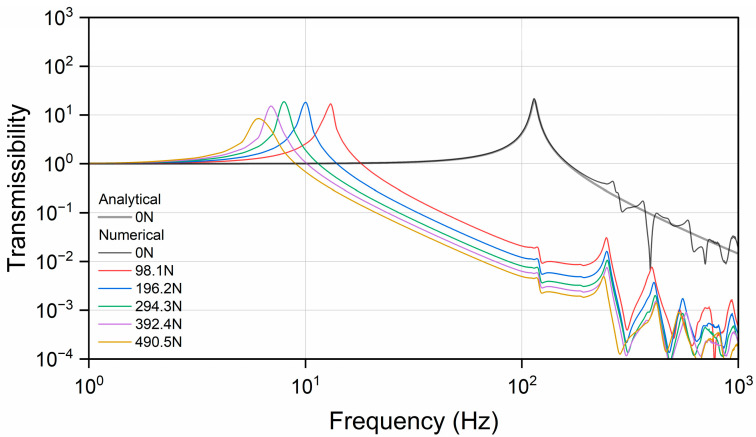
Analytical and numerical force transmissibility curve of result mount.

A series of dynamic simulations were conducted to investigate the transmissibility frequency response functions (FRFs) of the resilient mount with and without static preloads. Figure 10 shows the geometry with boundary conditions. The bolted joint was fully fixed in both translational motions (U) and rotational motions (UR). A rigid surface was attached to the top surface of the flange, and a reference node was created at the center of the top surface to apply a harmonic excitation. The dynamic simulations were conducted at 1 Hz intervals in the frequency range of 1–1000 Hz with preloads ranging from 0.0 N to 490.5 N. A structural damping ratio of 0.05 (5%) for the rubber element [27] is provided by the manufacturer. For the steel parts, where no other information is available, a structural damping ratio of 0.005 (0.5%) is used [28]. Figure 11 presents the force transmissibility both numerically and analytically, which is mathematically expressed as the ratio of the transmitted force F to the applied force $F_{i}$ as a function of frequency f:

(6) $$\frac{F}{F_{i}}=\sqrt{\frac{{f_{0}}^{4}+{\left(\eta f_{0}f\right)}^{2}}{{\left(f_{0}^{2}-f^{2}\right)}^{2}+{\left(\eta f_{0}f\right)}^{2}}}$$

(7) $$f_{0}=\frac{1}{2\pi }\cdot \sqrt{\frac{k}{M}}$$

where $f_{0}$ is the resonant frequency in Hz. η denotes the structural damping ratio. M and k represent the mass and stiffness, respectively. To validate the numerical model, the stiffness, defined as the ratio of force to displacement, was obtained from the static experiment on the resilient mount (as shown in Figure 8). However, due to the uncertainty of stiffness under preloading conditions, the analytical result is presented only for the case without preload. At certain frequencies, the system’s dynamics can no longer be accurately represented by a simple point mass and a perfect spring, resulting in complex resonant behavior. The numerical results show good agreement with the analytical results up to a certain frequency.

**Figure 12:**
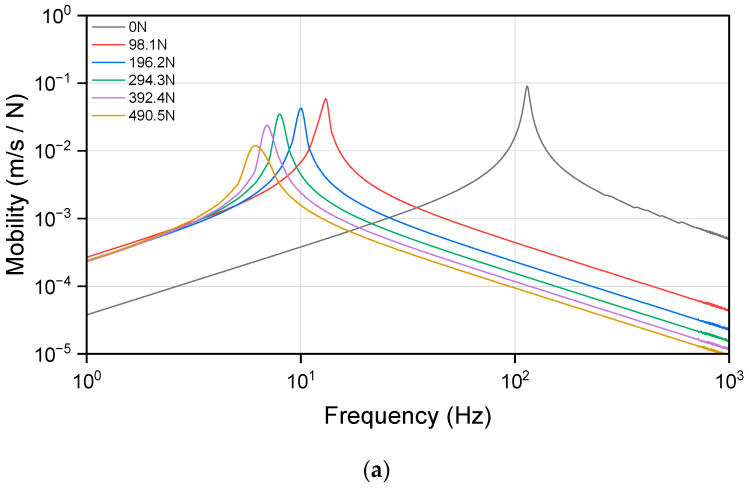

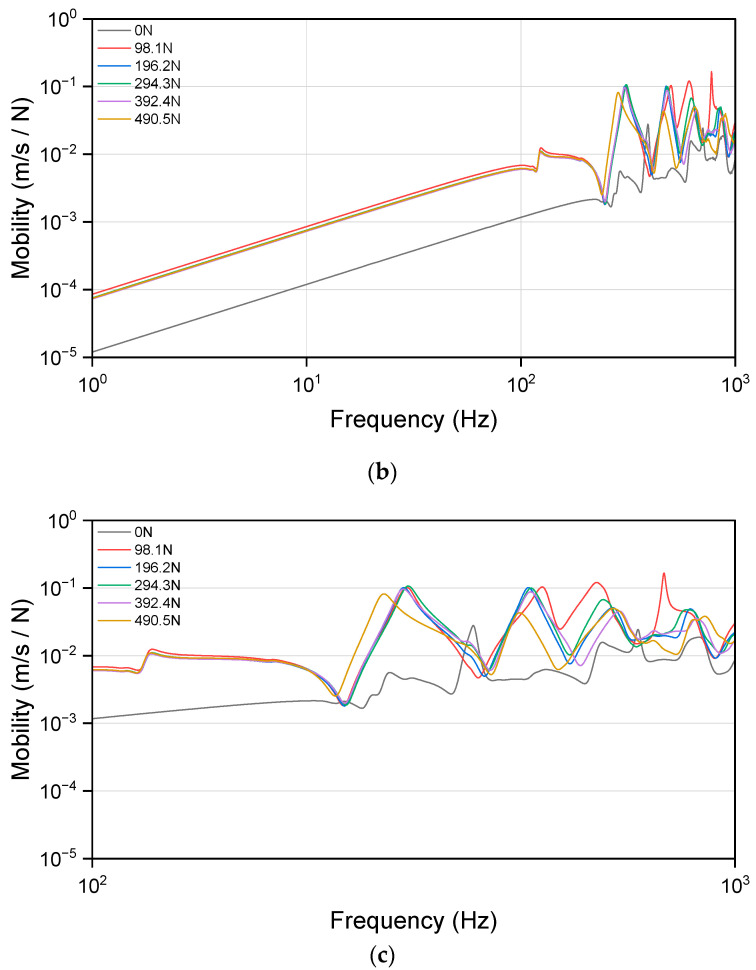
Dynamic mobility of resilient mount for different values of the preload (**a**) driving-point mobility, (**b**) transfer mobility from 1 Hz to 1 kHz, and (**c**) transfer mobility from 100 Hz to 1 kHz.

Figure 12 presents the mobility at both the driving and transfer points, defined as the velocity per unit force at the excitation point, along with the resonance peak for the resilient mount. Driving point mobility is measured at the location where the excitation force is applied, reflecting the dynamic response of the structure at this point and how it reacts to the applied load. In contrast, transfer point mobility is measured at the location where the load is transmitted through the structure, indicating the dynamic response of the structure as it transmits or deforms under the applied load. When preload is applied, the mobility curve and the position of the resonance peak shift significantly to a lower frequency. The resonance peak continues to shift to lower frequencies with increasing preload.

## 5. Conclusions

This study presents a comprehensive process for predicting the static and dynamic characteristics of resilient mounts used in naval ship applications. The key findings and contributions of this work are as follows:

- Quasi-static tests (uniaxial tension, planar tension, and equi-biaxial tension) were conducted to investigate the static behavior of the rubber material used in resilient mounts. These tests provided essential data for calibrating the Yeoh hyperelastic model.
- The Yeoh hyperelastic model was calibrated using the experimental data. The calibrated model was then validated through comparison with tension and compression tests performed on an actual resilient mount, showing good agreement between numerical simulations and experimental results.
- Modal analyses of the individual components (center plate, flange, and rubber element) provided insights into their frequency characteristics, with the rubber element showing significantly lower natural frequencies compared to the steel components.
- Frequency response analysis was conducted under various preloading conditions to investigate the dynamic behavior of the resilient mount. The results demonstrated that the transmissibility curves shifted to lower frequencies with increasing preload, highlighting the significant influence of the static state of stress on the dynamic properties of rubber-isolating elements.

This study underscores the importance of considering both static and dynamic characteristics in the design and analysis of resilient mounts. The demonstrated approach, combining experimental testing, material model calibration, and finite element analysis, provides a robust framework for predicting the behavior of these critical components under various loading conditions. The presented methodology offers a systematic approach for characterizing and modeling resilient mounts, providing valuable insights for optimizing vibration isolation systems in naval applications.

## Figures and Tables

**Table 1:** Strain energy density and error of experimental and numerical results for resilient mount.

Label	Strain Energy Density (J/m^3^)		Error (%)
Compression	Experiment	FEA	5.26
	237.25	224.76	
Tension	Experiment	FEA	1.94
	71.32	72.74	

## Data Availability

The original contributions presented in the study are included in the article, further inquiries can be directed to the corresponding authors.
